# Analysis of Pore Structure in Cement Pastes with Micronized Natural Zeolite

**DOI:** 10.3390/ma16134500

**Published:** 2023-06-21

**Authors:** Ionut-Ovidiu Toma, George Stoian, Mihai-Marius Rusu, Ioan Ardelean, Nicanor Cimpoeşu, Sergiu-Mihai Alexa-Stratulat

**Affiliations:** 1 Faculty of Civil Engineering and Building Services, The “Gheorghe Asachi” Technical University of Iasi, 700050 Iasi, Romania; 2 National Institute of Research and Development for Technical Physics, 700050 Iasi, Romania; gstoian@phys-iasi.ro; 3 Department of Physics and Chemistry, Technical University of Cluj-Napoca, 400114 Cluj-Napoca, Romania; mihai.rusu@phys.utcluj.ro (M.-M.R.); ioan.ardelean@phys.utcluj.ro (I.A.); 4 Faculty of Material Science and Engineering, The “Gheorghe Asachi” Technical University of Iasi, 700050 Iasi, Romania; nicanor.cimpoesu@academic.tuiasi.ro

**Keywords:** cement paste, micronized natural zeolite, pore structure, scanning electron microscopy (SEM), nuclear magnetic resonance (NMR)

## Abstract

The continuous development of urban areas around the world led to an increase in construction material use and demand, with concrete seeing significant market uptake. Although significant progress has been made to reduce the environmental impact of concrete, there is still a stringent need for improvement. One of the most widely used methods to reduce the environmental impact of the cement industry and the construction industry alike is the replacement of ordinary Portland cement (OPC) by supplementary cementitious materials (SCM). Aside from by-products of industry, SCMs could also come from natural sources. Taking into account the porous structure of zeolites and their contribution to the improvement of the mechanical and durability properties of cement-based materials, the analysis of pore structure in cement pastes incorporating micronized natural zeolite is deemed necessary. In this research, the OPC was replaced by zeolite in three different percentages: 10%, 20%, and 30% by mass. The evolution of pore structure was investigated by means of nuclear magnetic resonance relaxometry at the curing ages of 1, 7, and 28 days. The microstructure of cement pastes was assessed by scanning electron microscopy investigations at 1, 7, 14, 21, and 28 days. The obtained results show that smaller pore sizes are present in cement pastes containing zeolites during the first 7 days. However, at the age of 28 days, the reference mix exhibits a similar pore structure to the mix containing 10% micronized zeolite due to the presence of larger amounts of hydration products. Increasing the replacement percentage to 30% results in larger pores, as indicated by larger values of the relaxation time.

## 1. Introduction

**Figure 1:**
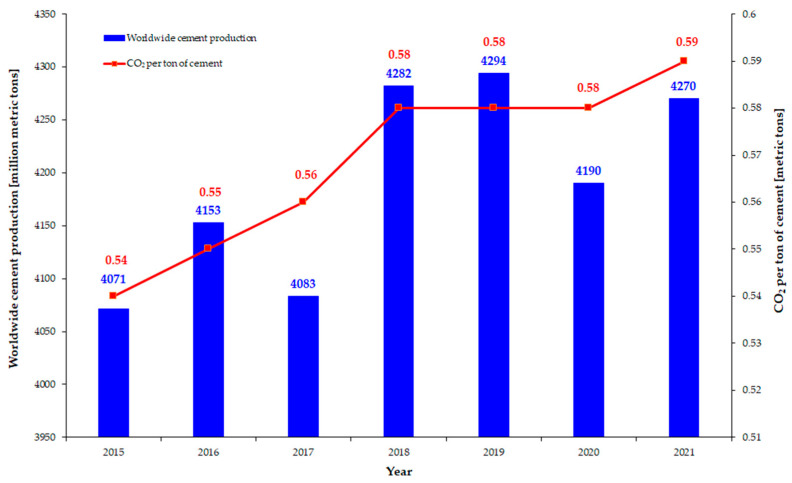
Evolution of worldwide cement production and associated CO_2_ emissions per ton of cement (data from [3]).

The continuous development of urban areas around the world led to an increase in construction material use and demand. The concrete market was forecast to reach 970 billion USD by 2030, with a compound annual growth rate (CAGR) of 4.7%. Consequently, cement production has to keep up, and it will reach a market value of almost half that of concrete by 2029, with a CAGR of 5.1%. Cement reacts with water to create a matrix, which binds the aggregates into a rock-like solid material known as concrete. It is the key component of normal to ultra-high-strength concrete. However, this comes at a cost in terms of greenhouse gas emissions (GHG), with the cement industry responsible for 7% of anthropogenic GHG [1]. Although the GHG associated with cement production has significantly decreased from 783 kgCO_2_/ton of cement [2], it is still a long way until net zero emissions are set as a target for 2050. Moreover, recent reports and statistics indicate a steady rise of 1.5% per year during the 2015–2021 time interval of GHG associated with cement production worldwide, as seen in Figure 1 [3]. Consequently, urgent measures need to be taken to reach the proposed intermediary goal of 472 kgCO_2_/ton of cement by 2030 [2], resulting in an annual 3% decrease in CO_2_ emissions. While the industry should focus on reducing the clinker/cement ratio, the implementation of innovative technologies for carbon capture and storage, the development of more energy-efficient kilns, and identifying alternative energy sources, the scientific community should intensify its research efforts to contribute to the development of new blended cements and/or cement-free binders [4,5].

One of the most widely used methods to reduce the environmental impact of the cement industry and the construction industry alike is the replacement of ordinary Portland cement (OPC) by supplementary cementitious materials (SCM). Due to their pozzolanic activity, the use of SCMs results in a long-term improvement of the mechanical and durability properties of cement-based materials. Fly-ash, a by-product of coal power plants, has been successfully used as a SCM due to the improvements it brings to cement-based materials in terms of strength [6], durability [7,8], and self-healing properties [9]. Ground-granulated blast furnace slag (GGBS) is another frequently used SCM due to its beneficial effect in terms of workability, long-term mechanical properties of mortar and concrete [10], improved pore structure [11], reduced shrinkage, and better durability [12]. The replacement of OPC by phosphogypsum, a by-product of fertilizer manufacturing, has also recently been recognized to have beneficial effects in terms of the elastic, mechanical, and durability properties of mortar and concrete [13,14,15]. However, in the case of phosphogypsum, a setting retarder is recommended.

Aside from by-products of industry, supplementary cementitious materials could also come from natural sources. Volcanic tuffs are a prime source for pozzolanic materials to be used as a partial replacement for OPC [16,17]. Zeolites, a naturally occurring volcanic rock, are known for their wide range of applications in agriculture, the petrochemical industry, cement industry, and, more recently, in nuclear waste repositories [18]. Being hydrated aluminosilicates, zeolites applications in cement-based construction materials have been intensively studied due to their pozzolanic activity [19]. Their porous structure enables zeolites to adsorb water and gradually release it during the curing period of cement-based materials, thus reducing autogenous shrinkage and providing a favorable environment for the long-term hydration of cement [19,20]. Zeolites strongly influence the rheological properties of cement pastes, with direct implications for the workability of fresh mortar and concrete [21]. Due to their open crystal structure coupled with a high specific surface area, they absorb some of the free water, thus reducing the workability of fresh cement-based materials (cement paste, mortar, and concrete) [22]. This may adversely affect the mechanical and durability properties of the resulting material if countermeasures are not considered. On the other hand, this apparent drawback becomes an advantage in the long run [19], with better hydration of the cement particles resulting in denser matrices and overall improved material properties. The main advantage consists in the fact that clinoptilolite natural zeolites do not swell or shrink during the process of absorption and desorption of water, being dimensionally stable. However, the porous structure of natural zeolites may adversely influence the pore structure of the cementitious matrix.

The pore structure is a very important parameter that influences the above properties and should, therefore, be correctly assessed. Recent studies have made it evident that the permeability and strength of cement pastes are governed by pore size distribution rather than porosity [23]. The smaller the pore size, the higher the compressive strength and the lower the thermal conductivity [24]. One of the most widely used methods for assessing the pore size distribution is mercury intrusion porosimetry (MIP). It involves careful sample preparation and is based on the high-pressure injection of mercury into the sample. At lower values of pressure, the mercury fills the larger pores but is unable to fill the smaller ones. Increasing the pressure results in mercury filling the smaller pores, both inter- and intra-particle pores [25,26,27]. There are, however, some limitations to using MIP to assess the pore structure of various media, such as cement pastes, mortar, and concrete. In one of the first studies to compare the results of using MIP with other assessing techniques, e.g., helium pycnometry, in hydrated Portland cement systems, it was concluded that significant results were obtained between the two methods [28]. The assumption was that mercury could not enter all the spaces, or closed pores, that are otherwise available to helium. Additional assessments of pore structure were conducted using methanol as a displacement fluid, and the obtained results confirmed prior findings. Later studies concluded that using MIP may not be entirely representative of the real pore structure. Even though the total porosity may have been correctly assessed, the pore size distribution varied significantly compared with other methods [29]. Various parameters need to be carefully considered when applying MIP to assess the pore structure, such as the varying contact angles between mercury and the solid part of the samples during the intrusion and extrusion stages, the effect of ink bottle pores entrapping mercury, and the pore connectivity effect [30]. A too high pressure applied may also lead to damage to the pore structure of the material [31].

Another technique for assessing the pore structure of porous media is low-field nuclear magnetic resonance (NMR) relaxometry [32]. NMR relaxometry of cement-based materials exploits the difference in relaxation times experienced by liquid molecules filling in different pore reservoirs. The main advantages of this technique over MIP are that it is completely non-invasive and does not require any special sample preparation. Furthermore, it can be applied even during the hydration process of cement-based materials [33]. This makes it possible to highlight the three types of pores inside cement-based mate-rials, during hydration and investigate the effects of different external parameters on them. However, precise absolute dimensions of the pores cannot be obtained without prior calibration of the pore surface relaxivity, and this can be achieved only by comparison with other techniques, such as MIP. The accuracy of the NMR relaxometry technique was highlighted in a recent study by comparing the results with the information acquired by MIP [34]. By using NMR relaxometry, the effect of calcium nitrate on the fresh and hardened properties of cement pastes was investigated and compared with the results extracted via X-ray diffraction (XRD) and scanning electron microscopy (SEM) [35]. It was also studied the influence of an accelerator on the pore structure development and the correlation with the strength of cement pastes made with CEM-I type of cement [36].

Taking into account the porous structure of zeolites and their contribution to the improvement of the mechanical and durability properties of cement-based construction materials, the analysis of pore structure in cement pastes incorporating micronized natural zeolite is of paramount importance. The OPC was replaced by micronized natural zeolite in three different percentages, 10%, 20%, and 30%, by mass, and the evolution of pore structure was investigated by means of low-field NMR relaxometry at the curing ages of 1 day, 7 days, and 28 days. The microstructure of cement pastes was assessed by SEM investigations. The SEM analyses offer valuable information about the internal structure of the material, e.g., the formation of ettringite, CH and CSH gels. Additionally, when conducted over longer periods of time, SEM investigations can provide information on the hydration processes and hydration products evolution that may explain the macro-properties of the material. The obtained results show that smaller pore sizes are present in cement pastes containing zeolites up to the age of 28 days, when the pore sizes in the cement pastes containing 20% and 30% micronized zeolites were slightly larger compared with the reference mix, as indicated by NMR relaxometry. Additionally, there seems to be a threshold, identified in this study as being 20% of cement replacement, above which the use of zeolites becomes detrimental from the point of view of the durability and strength characteristics of cement-based materials because it leads to an increase in the pore size.

## 2. Materials and Methods

### 2.1. Materials

The cement considered in this research was CEM II B-M (S-LL) 42.R, a rapid hardening composite Portland cement readily available on the market (HOLCIM), following the guidelines of SR EN 197-1:2011 [37]. The choice of this type of cement was based on the fact that it contains 65–79% clinker, thus contributing to lower GHG emissions. The remaining 21–35% of its composition consists of a mix of blast furnace slag and limestone. Another reason for considering this type of cement was the fact that it is the most commonly used type of cement in Europe. According to ref. [38], 47% of the cement sold in Europe in 2015 consisted of CEM II cement.

The natural zeolite powder used in the present research is based on Clinoptilolite (87–90%), being thermally stable up to 450 °C, has a pH value of 8.7, and has a mean particle distribution of 26.7 μm. The chemical compositions of the two materials, as supplied by the manufacturers, are shown in Table 1. According to ASTM C 618: 2022 [39], for the zeolite to be considered a natural pozzolan, the total content of SiO_2_ + Al_2_O_3_ + Fe_2_O_3_ should be at least 70%. Moreover, the SiO_2_/Al_2_O_3_ ratio greater than 4.0 confirms the Clinoptilolite type of the zeolite [40].

**Figure 2:**
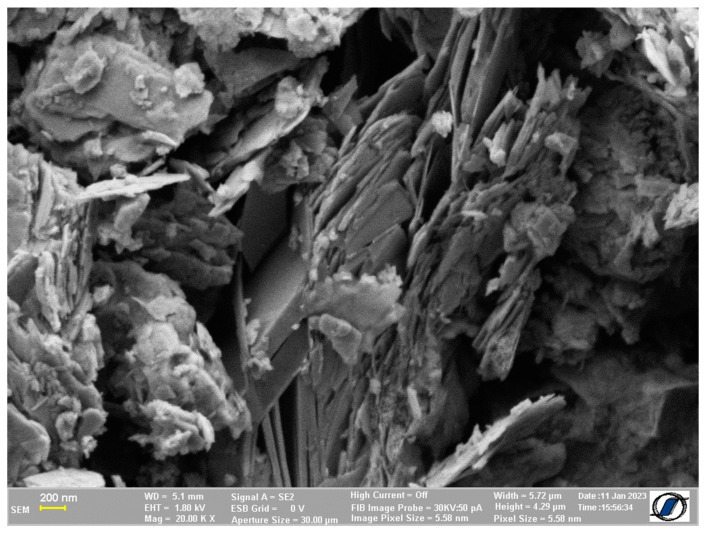
SEM image of clinoptilolite based natural zeolite.

The SEM image of the natural zeolite considered in this study is shown in Figure 2. The porous structure can be clearly seen. However, one advantage of the clinoptilolite-based zeolite is that it does not change its structure during the absorption or desorption of water and, therefore, is geometrically stable [19]. This is an important property since it does not create swelling or contractions in the cement matrix, which may lead to the formation of microcracks.

### 2.2. Methods

The mix proportions considered in this study are presented in Table 2. The water-to-binder ratio was kept at 0.6 for all mixes. The binder consisted of either Portland cement or Portland cement with natural zeolite. Three replacement percentages of CEM II OPC by clinoptilolite-based natural zeolite were selected: 10%, 20%, and 30% by mass of cement. The mixes were named to reflect the replacement percentage, e.g., ZP20 stands for paste with zeolite powder (micronized zeolite), ZP, replacing 20% of cement.

The pastes were poured into 10 mm diameter glass tubes and sealed to prevent loss of water.

The microstructure of the cement pastes, using a scanning electron microscope, was investigated at 1 day, 7, 14, 21 and 28 days of curing by means of a Carl Zeiss NEON 40EsB Cross-Beam system with thermal Schottky field emission emitter and accelerated Ga ions column.

The pore distribution in the cement pastes was assessed by means of the low-field nuclear magnetic resonance (LF-NMR) relaxometry technique using a Minispec MQ20 (Bruker, Heilderberg, Germany) instrument, operating at a proton resonance frequency of 20 MHz. The LF-NMR relaxometry allows the monitoring of the pore structure evolution and water consumption during the hydration process [32,33]. The Carr-Purcell-Meiboom-Gill (CPMG) [41,42] pulse sequence was applied to record the echo trains of each paste at the ages of 1 day, 7 days, and 28 days. The CPMG echo trains consist of 2000 echoes that were recorded after 32 scans, with an echo time of 80 µs and a recycle delay of 0.5 s. A numerical Laplace inversion algorithm applied to the recorded echo trains provided the relaxation time, T_2_, distributions [43].

The relaxation time can then be used to assess the dimension of the pores using the equation [44]:

(1) $$\frac{1}{T_{2}}=\rho \frac{S_{p}}{V_{l}}$$

where $S_{p}$ is the surface of the pore in contact with the confined liquid, $V_{l}$ is the volume of liquid inside the pore, ρ is the relaxivity, and T_2_ is the relaxation time. The relaxivity is a constant depending on the magnetic field induction, the pore surface, and the interaction between the molecules inside the pore and the surface of the pore itself (surface affinity). In case of saturated pores, when the liquid fills the entire pore volume, the following is considered $V_{l}=V_{p}$ in Equation (1). Assuming spherically shaped pores, Equation (1) becomes:

(2) $$T_{2}=\frac{1}{3\rho }\cdot R$$

where R is the radius of the pore. Note that, even if the relaxivity was unknown, the relaxation time distribution still provides information about the relative distribution of the pores.

Two distinct NMR measurements were performed at the age of 28 days. The first measurement considered the cement pastes in their initial condition, e.g., with water still present in the matrix. The second measurement was conducted on the samples after they had been fully immersed in cyclohexane for 4 h. Prior to the immersion stage, the samples were stored in a vacuum for 24 h in order to remove the free water from the capillary pores. Since cyclohexane has a longer relaxation time than water, this would result in a more clear separation between the pores from the gel hydration products and the capillary pores after the post-processing of the CMPG echo trains by numerical Laplace inversion [45].

Fresh properties of the cement paste, such as flowability and setting time, were also determined for each mix shown in Table 2. The flowability was assessed by means of a mini-slump test using a cone having a top and bottom diameter of 70 mm and 100 mm, respectively [46]. After the cone was lifted and the spread of the cement paste stopped, the average of two perpendicular diameters was considered. The initial and final setting times were determined in accordance with SR EN 196-3:2017 specifications [47].

## 3. Results

### 3.1. Flowability and Setting Time

**Figure 3:**
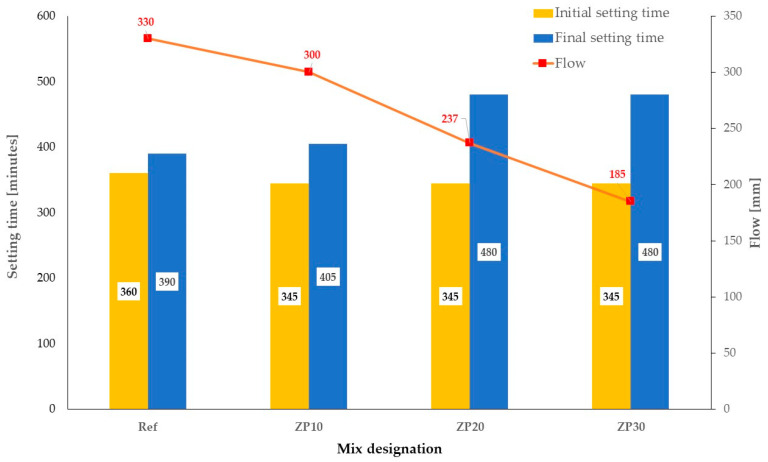
Flowability and setting times for the considered cement pastes.

The obtained results for the flowability of cement pastes and the initial and final setting times are presented in Figure 3.

It can be observed that the addition of micronized zeolite results in decreased values of the initial setting time compared with the reference mix. On the other hand, the final setting time value was larger compared with the one obtained for the reference mix by 3.85% for the ZP10 mix and 23.08% for ZP20 and ZP30 mixes. Increasing the replacement percentage of cement by micronized zeolite above 20% does not seem to influence the setting times values.

At the same time, replacing cement by micronized natural zeolite has a decreasing effect on the flow of cement paste. The final diameter of the ZP10 mix decreased by 9.09% compared with the reference mix, while increasing the replacement percentage to 20% and 30% resulted in 28.18% and 43.49% decrease, respectively. Similar decreasing trends were previously reported in the scientific literature [21,48].

### 3.2. SEM Analyses

Investigations on the material structure of the four cement pastes presented in Table 2 were conducted by means of SEM at the ages of 1 day, 7, 14, 21, and 28 days.

**Figure 4:**
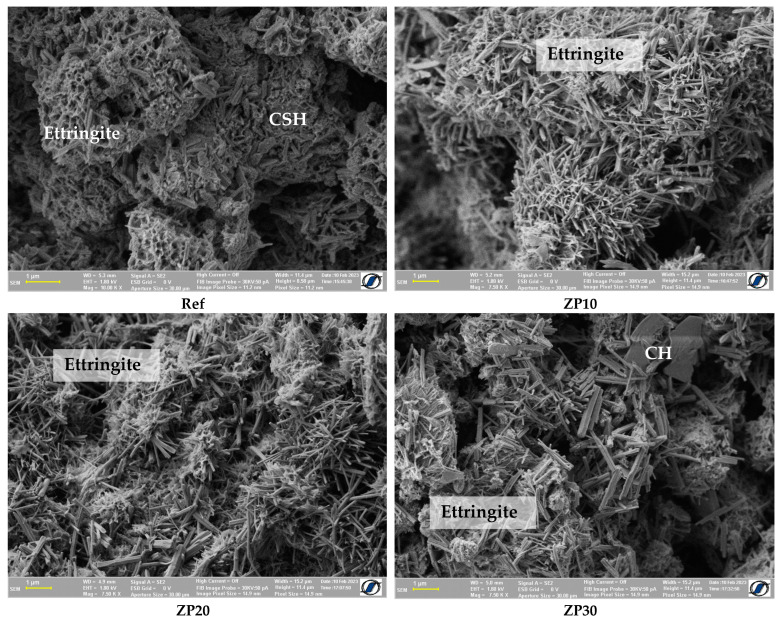
SEM micrographs of cement pastes at the age of 1 day.

Figure 4 depicts the SEM images one day after casting. A similar structure can be observed for all cement pastes, with ettringite being the main hydration product. The presence of calcium hydroxide (CH) hexagonal platelets can be observed for the ZP30 mix. Additionally, a more pronounced presence of calcium silicate hydrate (CSH) can be observed in the reference mix compared with other pastes. Calcium silicate hydrates, the main hydration product of OPC and the one responsible for the strength of the cementitious matrix, are heterogenous nanopore materials having an alternate structure of sheets containing calcium, oxygen atoms and silicate tetrahedra. CSH together with un-hydrated cement grains and other hydration products create a complex porous network containing intra-CSH pores, inter-CSH pores and capillary pores [44].

**Figure 5:**
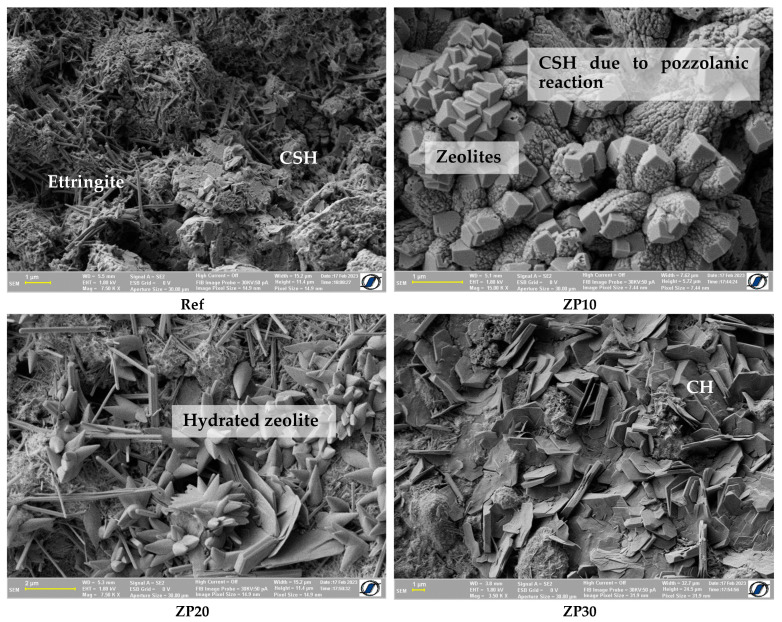
SEM micrographs of cement pastes at the age of 7 days.

The SEM images of the cement pastes at the age of 7 days are presented in Figure 5. Ettringite formations were still present in all cement pastes, but in a reduced amount. The zeolite particles [49] and the CSH gels due to the pozzolanic reaction of zeolites [50,51] could be clearly seen in the case of the ZP10 mix. Flower-like shapes of hydrated zeolite particles were observed in the ZP20 mix. Previous research works reported similar results for zeolites synthesized using aluminum dross and waste liquid crystal display glass powder [52]. ZP30 showed large amounts of CH formation.

**Figure 6:**
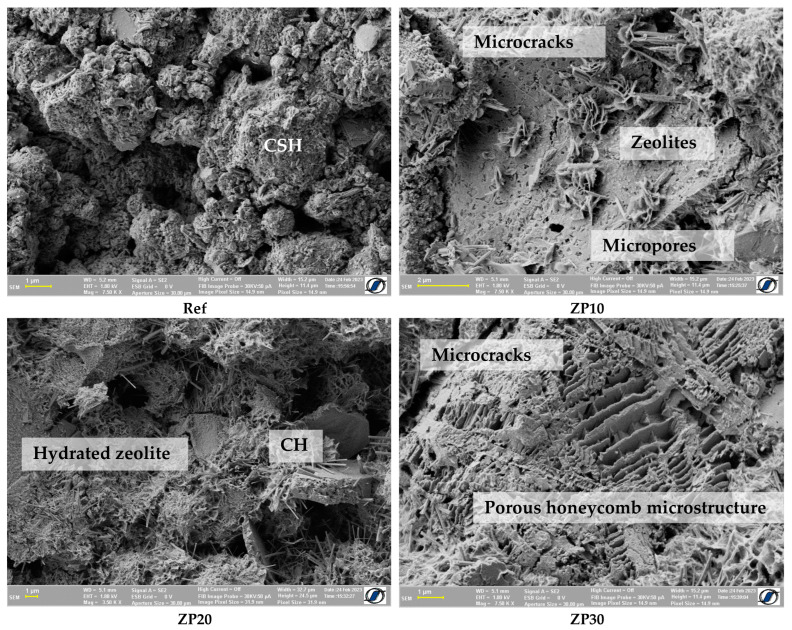
SEM micrographs of cement pastes at the age of 14 days.

The microstructure of all considered cement pastes at the age of 14 days is shown in Figure 6. The Ref mix showed quite an irregular structure, with “lumps” of CSH being separated by micropores. All micronized zeolite pastes exhibited a more compact structure, although similar microcracks and micropores to the reference mix could also be observed. Calcium hydroxide was still present in the zeolite-containing pastes, e.g., ZP20. This could be due to the pozzolanic reaction of zeolites, as reported in earlier research [53]. A porous honeycomb-like microstructure was observed for the ZP30 mix. This could be attributed to the porous structure of the zeolite combined with the high percentage replacement of OPC [54,55].

**Figure 7:**
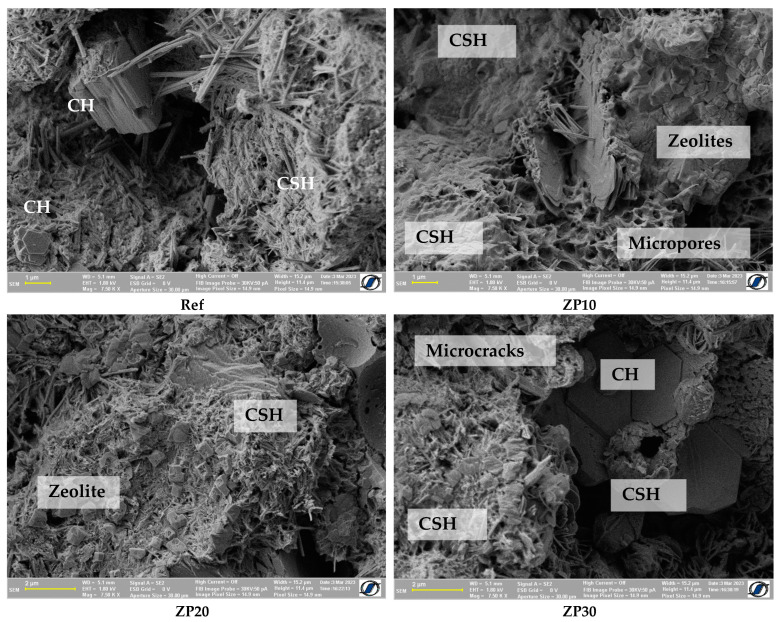
SEM micrographs of cement pastes at the age of 21 days.

More CSH formations could be detected at the age of 21 days for all cement pastes, as shown in Figure 7. The presence of CSH due to the pozzolanic reaction of zeolites [51,54] was detected in the ZP30 mix. Calcium hydroxide crystals were still present in the mixes. The porous structure of zeolites resulted in more micropores in the corresponding mixes, as can be seen from the SEM micrographs presented in Figure 7.

**Figure 8:**
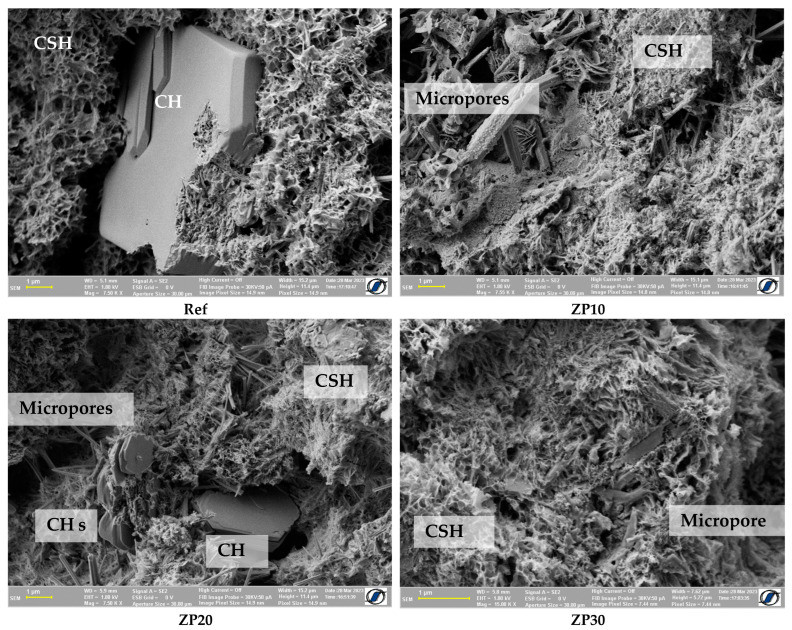
SEM micrographs of cement pastes at the age of 28 days.

Large amounts of CSH could be identified for all cement pastes at the age of 28 days, as shown in Figure 8. It can be observed that all considered mixes exhibited a denser structure compared with earlier ages with the number and size of micropores being smaller. Calcium hydroxide crystals could still be identified in the matrix. However, their dimensions were smaller in the case of ZP20 paste as compared with the reference mix. It can be assumed that the consumption of CH and its transformation into CSH at later ages were due to the presence of micronized zeolite acting as pozzolan [53,56]. In addition, taking into account the porous structure of the zeolite, which results in water absorption during the initial stages of the hydration process and water desorption at later ages, it could be hypothesized that the internal curing property of zeolites and the promotion of hydration reactions at later ages.

### 3.3. NMR Relaxometry

**Figure 9:**
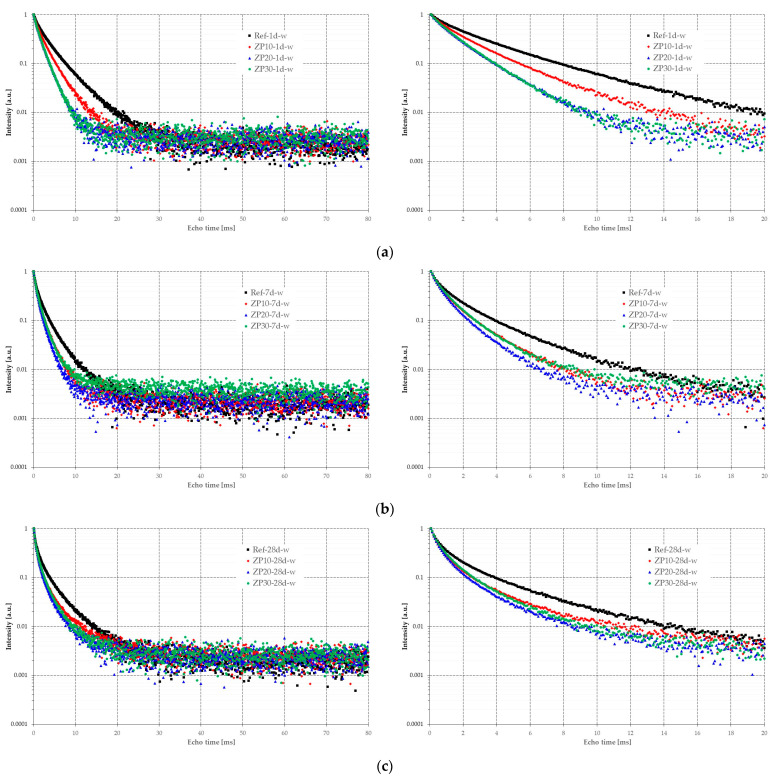
CPMG echo trains at different curing ages: full record (**left**); detail of initial decay slope (**right**). (**a**) 1 day curing; (**b**) 7 days curing; (**c**) 28 days curing.

Figure 9 shows the CPMG echo trains for all cement-based pastes at the ages of 1 day, 7 days, and 28 days. It should be pointed out that these results were obtained while there was still water present in the paste, meaning the hydration of cement was still going on. Consequently, the letter “w” was added to the designation in order to distinguish between different measuring conditions.

It can be observed that for each selected curing age, the initial slope of the echo trains of the pastes containing micronized natural zeolite was steeper than the slope for the reference mix. This could imply that pores with smaller diameters were formed inside pastes with zeolite. While at the age of 1 day, the ZP20 and ZP30 pastes showed almost identical decaying paths, starting from the seventh day, the ZP20 exhibited a steeper slope than all other pastes. The slope of the echo trains could also be a sign of the speed of the hydration processes taking place inside the paste, and the addition of micronized zeolite certainly has an influential role in promoting the formation of hydration products such as ettringite, CH, and CSH, as highlighted in Section 3.2. At the same time, a part of the micronized zeolite may act as a filler during the first day of the hydration process and reduce the volume of the voids between the cement particles. However, this requires more in-depth investigations in order to highlight the hydration process of the cement-zeolite binder during the initial stages.

**Figure 10:**
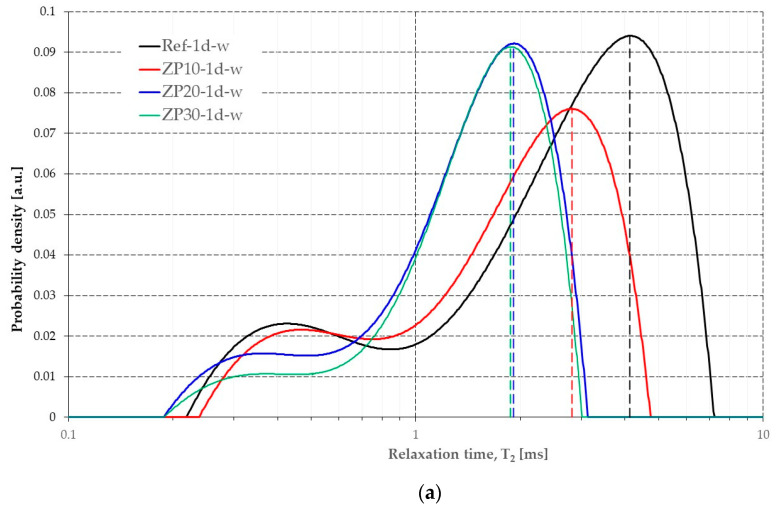

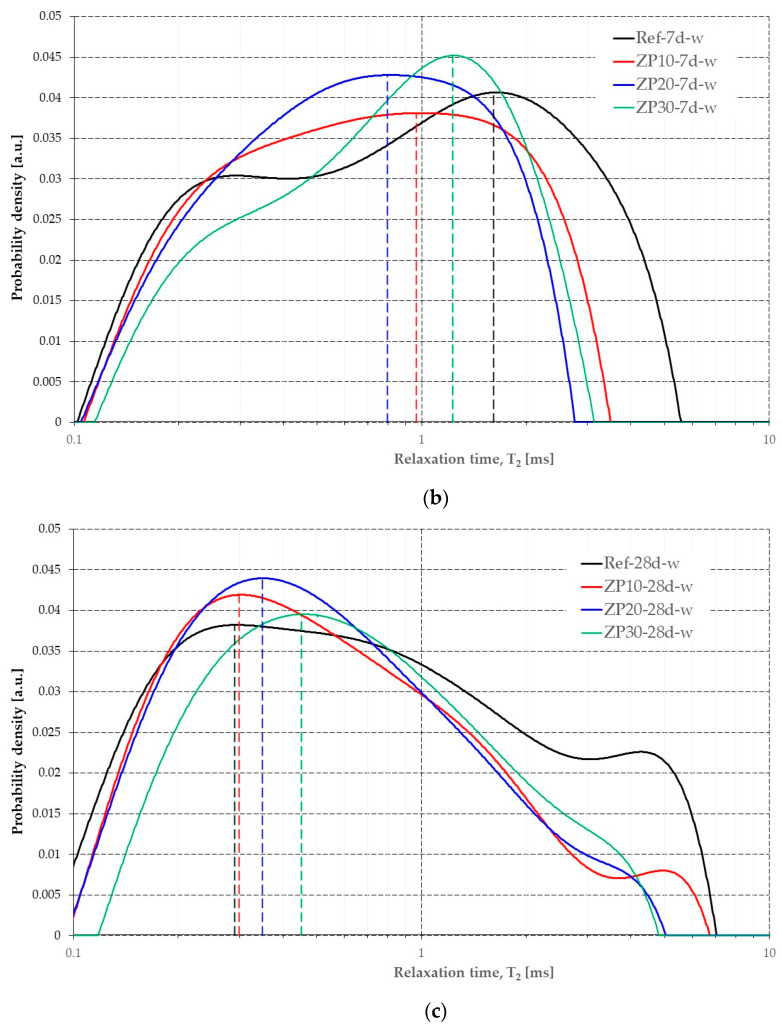
Relaxation time distribution of the considered cement pastes. (**a**) 1 day curing; (**b**) 7 days curing; (**c**) 28 days curing.

By applying a numerical Laplace inversion, the transverse relaxation times were obtained. Although the relaxivity was not assessed at this stage of the research, the pore size distribution could be discussed in a more general manner based on Equation (2). The change in the values of the transverse relaxation time for the considered pastes is presented in Figure 10.

The relaxation time T_2_ corresponding to the larger peaks in Figure 10 could offer qualitative information on the pore size distribution. Lower values of T_2_ mean smaller pore sizes, according to Equation (2). The shape of the graph changes from 1 day of curing to 28 days of curing because the pore structure changes due to the hydration reaction of cement particles coupled with the pozzolanic reaction of micronized zeolites. Therefore, larger pores that were initially filled with water gradually change their dimensions because more volume is occupied by the hydration products. The shift in T_2_ values towards the left at early ages of 1 day and 7 days means a smaller pore size for pastes containing micronized zeolites. At the age of 28 days, however, the Ref mix showed a smaller pore size structure compared with the mixes with micronized zeolite.

## 4. Discussion

The addition of micronized zeolite resulted in increased friction between the particles in the paste due to small particle size of zeolites and their higher surface area, which resulted in decreased flowability. Similar results were previously reported in the scientific literature, although for smaller water/binder ratios [21,48]. Furthermore, it should be pointed out that zeolites tend to absorb part of the water during the mixing procedure and early stages of cement hydration only to gradually release it at later ages [19].

The high water/binder ratio would result in a higher degree of hydration of the paste, up to 82% [57], which would result in larger amounts of hydration products. The presence of large amounts of CH and CSH at the ages of 21 and 28 days was highlighted by means of SEM investigations, as shown in Figure 7 and Figure 8.

The results obtained from NMR relaxometry are an indication of the pore structure of the cement pastes, Figure 10. At the age of 1 day, most of the water is located in the inter-CSH pores, represented by the higher peak in Figure 10a. The lower peak, at lower values of T_2_, corresponds to the intra-CSH pores, which are of smaller dimensions. The obtained results are in line with previously published research [33,35]. The increase in the OPC replacement percentage by micronized clinoptilolite-based zeolite resulted in smaller pore dimensions in the cement paste, as indicated by the shift towards the left in the values of relaxation time. This is supported by the micro-structure of the cement pastes presented in Figure 4. Although more ettringite was present in the case of ZP pastes, the structure was denser compared with Ref mix.

At the age of 7 days, Figure 10b, there was no clear difference between the peaks associated to capillary pores and the intra-CSH pores represented by the relaxation time of water molecules, especially for ZP10 and ZP20 mixes. Only Ref and ZP30 mixes exhibited a slight tendency to differentiate between the two types of pores. While in case of Ref the hydration process is still ongoing, in the case of ZP30 mix it may be still in its initial stage due to the pozzolanic reactivity of zeolites [50]. The pastes containing zeolites in different replacement percentages of OPC exhibited smaller pore sizes which was highlighted by smaller values of relaxation time (Figure 10b).

At the age of 28 days (Figure 10c) most of the water in the capillary pores was consumed by the chemical reactions in the cement matrix. Due to the rapid hardening properties of the CEM II cement, the Ref mix exhibited a denser structure compared with the ZP mixes. This resulted in lower values for the relaxation time compared with the zeolite-containing mixes. However, taking into account the fact that a composite cement was used (Section 2.1) the presence of CH crystals could be observed (Figure 8) [58].

**Figure 11:**
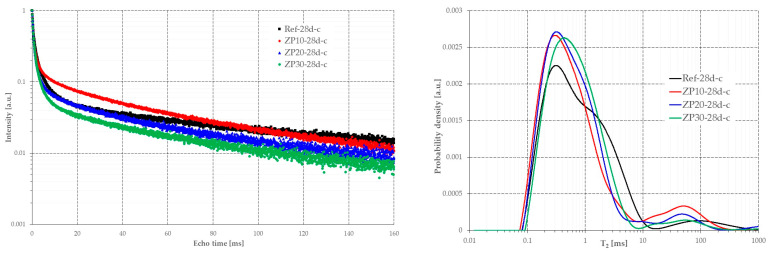
CPMG echo trains (**left**) and relaxation time distribution (**right**) of the considered cement pastes fully saturated with cyclohexane.

As previously mentioned, additional NMR investigations were conducted at the age of 28 days, with the samples being fully saturated with cyclohexane. The cement paste samples were placed in a vacuum for 24 h to remove the water from the inter-CSH pores and capillary pores. The water from the intra-CSH pores could not be removed without damaging the sample. The CPMG echo-trains were obtained by applying the same measuring parameters as described in Section 2.2. The obtained results are presented in Figure 11.

The obtained results are in line with the ones presented in Figure 9 and Figure 10 when the samples were still fully or partially saturated with water.

**Figure 12:**
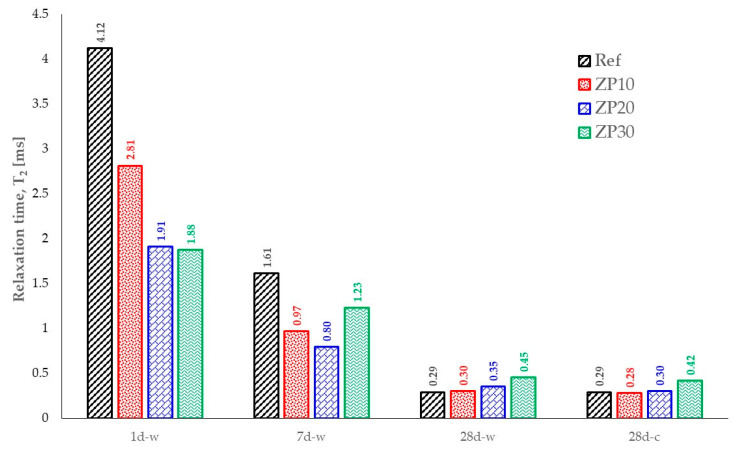
Time dependency of relaxation time for the considered mixes.

The evolution of the relaxation time, T_2_, for all cement pastes at all considered ages is summarized in Figure 12. It can be observed that at early ages, 1 day and 7 days, the Ref mix exhibited larger values of the relaxation time, meaning larger pores were present in the matrix compared with ZP mixes. This fact changed at the age of 28 days when ZP mixes exhibited slightly larger values of T_2_ compared with the Ref mix. Taking into account the small differences of 3.4% and 3.5; for the ZP10 and ZP20 mixes, respectively, it can be considered that the pore size distribution was almost similar between the mixes.

On the other hand, a 43.7% increase was recorded for the ZP30 mix compared with Ref mix. This could be attributed to the larger replacement percentage, 30%, of OPC by micronized zeolite. Taking into account the pozzolanic property of natural zeolite coupled with the use of blended cement, some of the chemical reactions in the cement paste would be slowed down [59], resulting in a more porous structure. Previous research showed that increasing the replacement percentage of blended OPC by natural zeolite resulted in smaller values of density, flexural strength, and compressive strength in mortars at an early age [60].

The results in terms of relaxation time obtained using cyclohexane were similar to those obtained on samples saturated with water. The same tendency in pore structure was observed in the case of cyclohexane-saturated samples.

**Figure 13:**
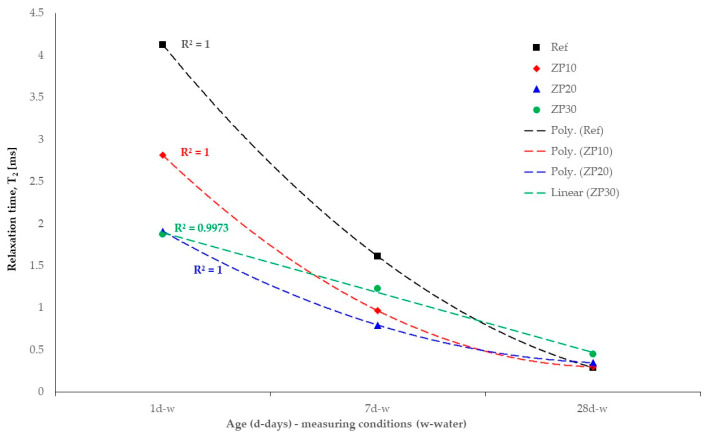
Trend of relaxation time values.

The decreasing trend of relaxation time values for the considered mixes is presented in Figure 13. While Ref, ZP10 and ZP20 mixes showed quadratic variation (2nd degree polynomial) of the relaxation time values with curing age, the ZP30 mix exhibited linear dependency. This could be attributed to slower chemical reactions in the paste due to the high percentage of OPC replacement by zeolite and taking into account the type of cement used in this research.

The speed of hydration product formation in the cement pastes could be indicated by the slopes of the decaying curves. Ref mix shows the steepest decay compared with ZP10 and ZP20 mixes [55]. The hydration reaction took place at a higher speed, and the pore sizes decreased faster. This is in line with the SEM micrographs presented in Figure 4, Figure 5, Figure 6, Figure 7 and Figure 8. There were no notable differences in the microstructure of Ref mix from day 7 to day 28 of curing.

As seen from Figure 13, increasing the zeolite percentage results in a slower decrease in the pore size of the matrix, as shown in Equation (2). Moreover, the increase in the zeolite percentage results in smaller pore diameters at early ages, 1 day and 7 days, compared with the reference mix. At the age of 28 days, however, the ZP30 mix exhibited larger pores, as indicated by the results presented in Figure 11.

## 5. Conclusions

The paper presents the results obtained during an experimental program aimed at assessing the pore structure in cement pastes with micronized natural zeolites used as cement replacement. The considered percentage replacements were 10%, 20%, and 30% by mass of cement. The pore structure was qualitatively assessed by means of NMR relaxometry, and the obtained results were compared and validated using scanning electron microscopy. Based on the obtained results, the following conclusions can be drawn:

1. The gradual generation of CSH gels with curing age is rendered evident by means of SEM analyses. Moreover, CSH formations due to the pozzolanic reaction of micronized zeolite are also identified. The microstructure of cement pastes becomes more compact and denser with curing age. At the same time, fewer micropores can be identified. A replacement of OPC by natural zeolites in the range of 10–20% by mass of cement is also reported in the scientific literature as leading to the highest improvements in terms of durability properties and long-term strength values of cement-based materials. The microstructure analyses confirm the fact that smaller pores are obtained in cement pastes with a 10% and 20% replacement of OPC by natural zeolite. Increasing the replacement percentage to 30% results in larger pores due to a higher amount of water being absorbed and stored in the porous structure of zeolites.
2. The NMR relaxometry technique is used to qualitatively assess the pore structure of the cement pastes. At an early age, e.g., 1 day, there is a clear distinction between the inter- and intra-CSH pores. The presence of water molecules in the former pores results in larger relaxation time values, whereas the latter yield lower values of the relaxation time. The replacement of OPC by micronized natural zeolites results in a shift towards lower values of relaxation time, which means smaller pores in the cementitious matrix. While at early ages the ZP20 and ZP30 pastes have similar pore structures, as a result of the very close values of relaxation time corresponding to the mixes, at the ages of 7 days and 28 days the values of T_2_ corresponding to the ZP20 mix are closer to the ones corresponding to the ZP10 mix. This could indicate that there might be a threshold of cement replacement by micronized zeolite above which the effect might be detrimental in terms of pore structure.
3. At the age of 28 days, the pore structures of the Ref, ZP10, and ZP20 mixes are similar. The T_2_ values of the ZP10 and ZP20 mixes are slightly larger than the one of Ref mix by 3.4% and 3.5%, respectively. The ZP30, however, registers a 43.7% increase in the value of relaxation time, indicating the presence of larger-diameter pores. Similar results are obtained when saturating the samples with cyclohexane. The pozzolanic nature of zeolite, coupled with the use of blended cement, leads to slower development of hydration products, e.g., CSH, as demonstrated in the scientific literature. Higher percentages of zeolite result in lower amounts of available CH, leading to decreased formation of CSH gels due to secondary pozzolanic reactions. As a result, the decreased availability of CH results in a lower amount of water needed for chemical reactions, and, therefore, the water absorbed by zeolite during the early stages of hydration remains trapped in the pores. This can have detrimental effects on the durability and strength development of cement-based construction materials in the long run.
4. Although the pore structure of cement pastes containing zeolites as a replacement for OPC in different percentages can be assessed by means of NMR relaxometry, a more in-depth analysis is required to determine the relaxivity constant. The results obtained by NMR should be confirmed by other assessment techniques such as computer tomography (CT).

## Figures and Tables

**Table 1:** Chemical composition of cement and natural zeolites (expressed in %).

	CaO	SiO_2_	Al_2_O_3_	Fe_2_O_3_	MgO	SO_3_	Na_2_O	K_2_O	Cl
CEM II	58.49	16.32	4.83	2.24	1.39	2.85	0.34	0.46	0.035
Zeolite	2.86–5.2	68.75–71.30	11.35–13.10	1.90–2.10	1.18–1.20	-	0.82–1.30	3.17–3.40	-

**Table 2:** Mix proportions.

Mix	Binder		Water/Binder
	CEM II	Micronized Zeolite	
	[%]	[%]	[%]
Ref	100	-	0.6
ZP10	90	10	
ZP20	80	20	
ZP30	70	30	

## Data Availability

Not applicable.
